# The Acute Care of Chronic Pain Study: Perceptions of Acute Care Providers on Chronic Pain, a Social Media-based Investigation

**DOI:** 10.7759/cureus.2399

**Published:** 2018-03-30

**Authors:** Eric Chen, Daniel Tsoy, Suneel Upadhye, Teresa M Chan

**Affiliations:** 1 Faculty of Health Sciences, Department of Medicine, Division of Emergency Medicine, McMaster University

**Keywords:** chronic, pain, attitudes, emergency physician, chronic pain, nurse, paramedic, pain management, online survey, physician assistant

## Abstract

Introduction

The diagnosis of chronic pain involves symptoms of pain of various etiologies lasting longer than six months. The prevalence of chronic pain in society ranges from 19% to 31% in North America. While chronic pain patient perceptions on the care provided to them in the Emergency Department (ED) have been studied, there has not been significant attention given to the attitudes of acute care providers towards these patients.

Methods

We utilized online questionnaires disseminated on Twitter, Facebook, Reddit, and emergency medicine blogs to gauge care provider attitudes of chronic pain patients. Survey respondents included ED physicians and their trainees, ED nurses and nurse practitioners, paramedics, and physician assistants.

Results

Responses revealed numerous factors impacting care provider dissatisfaction with treating chronic pain in the ED; significant factors included the lack of longitudinal care and inappropriate medication of chronic pain resulting in dependency. We found that additional chronic pain-specific training was associated with increased care provider confidence in the treatment of chronic pain. Practice patterns were found to be varied, with half of the respondents stating that chronic pain should be medicated acutely.

Conclusions

We conclude that acute care provider dissatisfaction with chronic pain treatment is multifactorial in origin and that confidence in the acute treatment of chronic pain can be improved with chronic pain-specific training.

## Introduction

Chronic and recurrent pain is a highly prevalent medical condition which negatively impacts the quality of life. The condition affects up to 18.9% and 31% of Canadian and American adults, respectively [1]. Chronic pain is a significant contributor to emergency department (ED) visits, with the reported prevalence of underlying chronic pain conditions accounting for up to 40% of pain-related ED visits [2]. Despite the high frequency of chronic pain-related ED visits, the ED remains a suboptimal environment for the treatment of chronic pain due to the inconsistency in care provided for patients inherent in them seeing a different practitioner each visit, as well as the financial pressure on healthcare resources that arises from the vastly higher cost of an ED visit compared to a general practitioner visit [3-4]. Chronic pain patients' experiences with ED care has been investigated, with a literature review of managing chronic or recurrent pain in the ED identifying three main themes of uncertainty: patient expectations, patient-provider barriers to care, and a lack of ED strategies to manage these patients [5]. Further studies suggest that ED patients find deficits in physician communication in pain management and opioid risk options [6]. A recent mixed-methods study performed by Poulin et al. have concluded that chronic pain patients’ dissatisfaction with the care offered to them stems from the rapid pace of assessment and treatment in the ED being suboptimal for the effective treatment of chronic pain [7]. Given the high prevalence and associated cost of ED visits related to chronic pain, it is worthwhile to examine potential care provider factors contributing to the suboptimal care and established patient dissatisfaction. To this end, we conducted a study to explore acute care provider training and attitudes towards the treatment of chronic pain patients.

Past surveys of physician attitudes suggest that staff, residents, and medical students hold negative views of patients suffering from chronic pain [8]. Many are reluctant to prescribe analgesics to these groups in the ED [9-10]. Reasons for this include the short-term nature of physician-patient relationships in the ED, concerns regarding patient addiction, care provider knowledge of pain management and comfort with pain management techniques, and a fear of being audited by a regulatory body [9-12]. In particular, knowledge deficits and misconceptions regarding pain treatment by healthcare professionals have been demonstrated consistently within prior research [13-15].

Additionally, there is great variability in the ED care provided for patients with chronic pain [16]. Past literature has suggested that this may be influenced by the attitudes and level of training of ED care providers [17]. However, quantitative measurements of these factors and how they affect daily practice in the ED is limited. This study aims to characterize the effect of additional chronic pain training on self-perception of ability to manage the condition, differences in prescriber vs non-prescriber attitudes towards medication, and finally, general attitudes of acute care providers of patients with chronic pain.

## Materials and methods

Study design

Inclusion criteria for participation in this survey were all acute care providers licensed by a local regulatory body and their trainees. These included emergency medical doctors (MDs), resident physicians, medical students, registered nurses (RNs), physician assistants (PAs), nurse practitioners (NPs), and paramedics. A 26 item instrument was developed by the investigators (Appendix A). The instrument included questions assessing baseline demographic data from the care provider, as well as attitudes towards chronic pain patients and patients who chronically use analgesics. Facebook, Reddit, Twitter, and the Canadian emergency medicine blog, CanadiEM, were used for recruitment as outlined in Table 1. Study recruitment by Twitter and Facebook was performed in accordance with previously reported guidelines [18-19].

**Table 1:** Dissemination of the Acute Care of Chronic Pain Study Across Various Social Media Platforms

Study recruitment method	Description
CanadiEMblog post	Public invitation to participate in the survey in a blog post displaying the survey poster
Twitter	Invitations to participate in the survey sent to self-identified acute care providers, journals, and interest groups on Twitter via tweets containing the survey link
Facebook	Personal Facebook profiles were used to send survey links via Facebook messages and page shares to resident, physician, and paramedic groups
Reddit	Posts containing the survey link were made to sections (“subreddits”) of the website with a special interest in areas such as medicine, emergency medicine, and nursing to maximize the number of potential participants.

Participants who clicked on the link from any of the social media websites were redirected to the same survey website. The protocol used is similar to the one used in a prior study [20]. Participants who visited the survey website were asked to complete the survey in private. Prior to opening the survey, informed consent was obtained from study subjects with an introduction explaining the purpose of the study in greater detail than listed on each of the social media websites. In addition, potential risks, as well as steps to protect study participants, were explained. We used the online survey tool, Google Forms, over a secure encrypted connection to administer the survey to participants. To further protect the participants, internet protocol addresses were not collected. All personal identifying information was removed from the results prior to analysis. Demographic data reported by participants was used by the authors to determine eligibility to participate in the study.

Research ethics approval for this study was obtained from the Hamilton Integrated Research Ethics Board (HIREB #2017-1814).

The enrollment period ranged from 0000 EST, December 1, 2016 to 0000 EST, August 31, 2017. Survey respondents were offered a chance to win one of four $250 CAD Amazon.ca gift cards as an incentive to complete the study.

Data analysis

Following the end of the enrollment period, the survey was closed and data analysis was conducted. Sentence-based responses were analyzed qualitatively with the use of a frequency table. Two authors (EC, DT) reviewed the dataset independently and resolved conflicts by a consensus building process. Responses to Likert scale questions were analyzed quantitatively. All calculations were performed with Microsoft Excel and the Microsoft Statistical Analysis Toolpak (Microsoft Corp., Redmond, WA).

## Results

Demographics

The number of social media engagements are outlined in Table 2.

**Table 2:** Engagement Metrics with the Acute Care of Chronic Pain Study Across Various Social Media Networks

Technique	Number of Engagements
CanadiEM Blog Post	278 Visits
Twitter	129 Tweets, 103 Retweets, 1280 Tags, 49 Followers
Facebook	47 Messages sent, 20 Posts
Reddit	3 posts, 5 comments

The survey has received 201 responses. Twenty-one respondents were trainees and 180 were licensed care providers. Of the licensed care providers, 84 were ED MDs, 50 were ED RNs, 34 were paramedics, and 9 were PAs. Ninety-six percent of respondents stated that English was their primary language or language of practice.

Ninety-five percent of physicians were licensed in emergency medicine. Within the nursing group, 66% were certified ED/Trauma nurses, 8% were NPs, and the remaining 26% of nursing respondents stated that they had other qualifications (e.g. nurse manager, nurse educator, etc).

Respondents’ locations of practice were distributed across 13 countries (Australia, Brazil, Canada, Costa Rica, Denmark, Greece, Portugal, Romania, Saudi Arabia, South Africa, Switzerland, United Kingdom (UK), United States (US)), with 90% of respondents located within the US and Canada.

Acute care provider training in chronic pain

Eighty-one percent of respondents did not receive additional training in the treatment of chronic pain. Of those who received additional training, modalities varied widely and included, but were not limited to, supplementary lectures on chronic pain care, participation in online and in-person chronic pain care courses, and attending conferences with an incorporated chronic pain curriculum (Table 3).

**Table 3:** Frequency of Additional Chronic Pain Training Modalities for Acute Care Providers

Training type	Frequency
Lectures	8
Classroom and online	3
Online workshop	1
Clinical semester in pain medicine	1
Continuing Medical Education (CME) courses	3
Computer-based training module	7
Conference	2
Didactic +/- exam	2
Master's degree in end-of-life care	1
Residency and state-mandated opioid training	1
Undergraduate university course	1
Predoctoral Osteopathic Manipulative Medicine Fellowship	1
Self-directed	1
Unspecified	5

Thirty-five percent of respondents stated that they did not feel adequately trained to treat chronic pain. Provider confidence in their ability to treat chronic pain was assessed on a five-point Likert scale (with a rating of 1 indicating "not at all confident", and a rating of 5 indicating "very confident"). Participants who received additional training in chronic pain felt significantly more confident in the management of chronic pain in the ED (mean confidence in treatment = 3.8) than participants who did not receive such training (mean confidence in treatment = 2.9; p < 0.001).

Familiarity and practice patterns

Eighty-three percent of respondents stated that they do not utilize guidelines in the treatment of chronic pain. The remaining 17% of respondents stated that they used guidelines in the treatment of chronic pain, examples of which include guidelines from the American College of Emergency Physicians, the Canadian College of Family Physicians, and the World Health Organization.

Familiarity with pain syndromes varied widely. Ninety-eight percent of participants were familiar with fibromyalgia, neuropathic pain, and inflammatory pain. Seventy-five percent of respondents were familiar with chronic myofascial pain.

Attitudes towards medication for chronic pain

**Figure 1:**
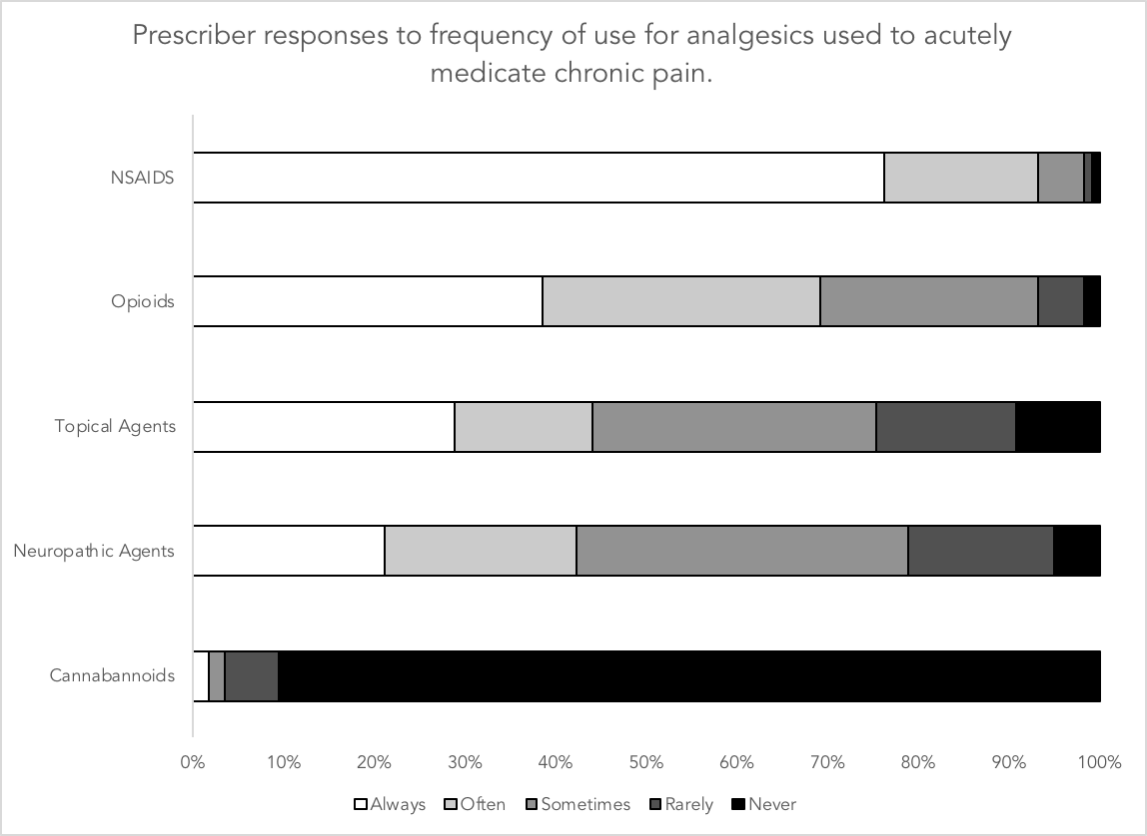
Prescriber responses to frequency of use for analgesics used to acutely medicate chronic pain NSAIDs: nonsteroidal anti-inflammatory drugs

Fifty percent of respondents stated that physicians should not prescribe analgesics, including opioids, for chronic non-cancer pain in the ED. Respondents with prescribing privileges (MDs, PAs, and NPs) were asked whether they prescribe NSAIDs, opioids, neuropathic medications, topical medications, and cannabinoids when clinically indicated in the ED. These results are illustrated in Figure 1. Prescribers responded “always” or “often” to the following: NSAIDs (93%), opioids (69%), topical medications (44%), neuropathic agents (42%). Two percent of prescribers responded with “always” or “often” when asked about whether they prescribe cannabinoids.

Providers were asked to rate their agreement with the statement that “Physicians should prescribe analgesics, including opioids, for chronic non-cancer pain” on a five-point Likert scale (with a rating of 1 indicating "Completely disagree", and a rating of 5 indicating "Completely agree"). Providers with prescribing privileges (staff physicians, residents, nurse practitioners, and physician assistants) were significantly less likely to agree that medicating for chronic pain in the ED is appropriate (mean agreement = 2.4) compared to non-prescribing care providers (nurses and paramedics; mean agreement = 2.9; p = 0.012).

Attitudes

Of the qualitative responses to the question of what words and/or phrases come to mind when describing a patient with chronic pain, several general themes emerged (Table 4). Responses expressing concern regarding systemic problems with the treatment of chronic pain included the requirement for longitudinal care not available in the ED (16 responses), inappropriate treatment from primary care (27 responses), and drug-seeking behavior or dependency (32 responses). Participants also voiced negative perceptions of chronic pain in the emergency department, including the words difficult (22 responses) and frustrating (16 responses).

**Table 4:** Predominant Themes in Care Providers’ Answers to the Question of “What Words and/or Phrases Come to Mind When Describing a Patient with Chronic Pain”

Theme (Number of responses)	Exemplar Quotes
Negative perceptions of chronic pain (61 responses)	“Difficult to treat”, “Frustrating”, Chronically undertreated and highly dissatisfied”
Dependency on opioids (32 responses)	“Opiate-positive”, “Tolerant”, “Dependent”
Inappropriately treated (27 responses)	“Nonnarcotic options”, “too reliant on pain medications instead of alternatives, such as exercise”
Frustration around lack of primary care (16 responses)	“Never an emergency”, “Tired of waiting to be seen by primary care provider”

## Discussion

Through social media promotion, our survey reached an appreciable cross section of acute care disciplines with varying degrees of training in pain management. 

Our survey revealed substantial differences in training, attitudes, and treatment of chronic pain. A minority of respondents stated that they had training specific to chronic pain (19%), which was delivered in varied formats, including lectures, conferences, and computer-based modules, among others. Despite this, a majority (65%) of providers stated that they believe they had sufficient training to adequately treat chronic pain, a finding which is consistent with medical literature regarding provider confidence in chronic pain management [17]. Furthermore, the self-reported familiarity with chronic pain syndromes was high, with 98% of providers claiming familiarity with fibromyalgia, neuropathic pain, and inflammatory pain, and 75% of providers reporting familiarity with chronic myofascial pain. Provider confidence in their training and existing familiarity with pain syndromes may be the reason that a minority of subjects sought out further courses in pain management. However, we found that additional training in chronic pain management, regardless of the format, was significantly associated with an increase in respondent confidence in providing treatment. The lack of training in chronic pain care may also explain the low rate (17%) of provider utilization of treatment guidelines via a lack of confidence in chronic pain treatment, as past studies have shown that provider confidence in providing treatment is positively correlated with increased utilization of treatment protocols and increased confidence in identifying patients at risk of misuse [21-22]. Additional training on chronic pain care via modalities, such as educational lectures, workshops, or CME courses, may, therefore, increase care provider utilization of treatment guidelines and improve the quality of care experienced by patients presenting to the ED with chronic pain exacerbations. Wilsey et al. explored barriers to ED chronic pain management and concluded that, although there is a general desire for ED physicians to treat chronic pain patients, time limitations and low triage category are common barriers to this activity [23]. In a related study, they also found that patients were more likely to believe that there was a physical component to their pain, physicians were more inclined to believe that patients seeking ED analgesia did not have a primary care physician, and both agreed that chronic pain treatment ED was a low priority [24].

We explored the opinions surrounding acute treatment of chronic pain using medications. Fifty percent of respondents indicated that acute treatment of chronic pain with analgesics, including opioids, was an appropriate management strategy. We conducted statistical analyses to investigate whether the opinion of prescriber respondents differed from the opinion of non-prescriber respondents. Our analysis revealed that the prescriber respondents felt medication with analgesics was overall a less appropriate strategy for the treatment of chronic pain compared to non-prescriber respondents. Despite this, when asked about acute treatment regimens administered to chronic pain patients, the majority of prescribers responded that they “always” or “often” prescribe NSAIDs (93%) and opioids (69%).

We were interested in investigating the reasons behind why prescribers overall have a negative opinion of medicating for chronic pain acutely. To this end, we turned to the results of our subjective, short-answer survey question of what words and phrases come to mind when describing a patient with chronic pain. A significant proportion of survey respondents included phrases which indicated frustration regarding the lack of primary care for patients with chronic pain, as well as frustration regarding care provider reliance on opioid treatment with subsequent dependence on opioids by chronic pain patients. It is possible that prescribers’ overall opinions against acute medication of chronic pain stem from frustration regarding the presentation of chronic pain patients, who ideally would be managed by their family physician, to acute care settings, as well as the prevalence of dependence in chronic pain patient populations. However, given the relative paucity of treatment regimens as effective as NSAIDs and opioids in the acute treatment of pain, many prescribers still resort to these medications when managing chronic pain patients.

The potentially harmful effects of medicating chronic pain acutely, especially with opioids, has been thoroughly investigated [25-26]. Prescribers who are of the opinion that chronic pain should not be acutely medicated may be trying to balance the benefit of treating the patients’ symptoms against the potential harm of medical intervention and may believe that appropriate longitudinal management would lead to optimization of care for these patients, as well as avoiding adverse side effects of medication.

The strengths of our survey include respondents' diversity in acute care disciplines. However, the total number of respondents is small relative to the size of the target population and over-represents respondents from the United States and Canada (90%), limiting the generalizability of our findings. Some weaknesses exist as inherent to the study being social media-based. Our ability to calculate a response rate is limited as we lack reliable data for the number of views for our postings. We also acknowledge the possibility of volunteer bias arising from differences in training and attitudes of acute care providers who chose to participate in the study, compared to those who chose not to participate in the study. Furthermore, we acknowledge the possibility that participants could have responded multiple times to the survey with the use of a second e-mail address. Finally, our survey population may over-represent populations who are active on social media compared to those who are not.

## Conclusions

The significant prevalence of chronic pain as the reason behind many patients' visits to the ED, as well as these patients' widespread dissatisfaction with the care they receive, encouraged our team to investigate care provider training and attitude as factors contributing to the inadequate quality of care. Our findings show that a significant proportion of acute care providers have minimal targeted training around the acute treatment of chronic pain and that this lack of training may contribute to potentially negative attitudes and varied practice patterns towards patients presenting to the ED with pain exacerbations. We hope that future studies may better examine the impact of increased chronic pain training on patient outcomes.

## Appendices

Appendix A – Survey

Demographic Information

1)      Are you a licensed and practicing acute care provider?

2)      Is English your first language or language of practice?

3)      Participant type:

a)      Physician – please specify specialty

b)      Nurse – please specify specialty or additional training (e.g. nurse practitioner)

c)       Paramedic

d)      Occupational therapist (OT)/physical therapist (PT)

e)       Respiratory therapist

f)        Social Worker

g)      Other allied health profession – please specify

h)      Student/trainee – program and specialty, if applicable

4)       If your response to question 3 was "h) Student/trainee", please specify how many years you have progressed in your training

a)      1

b)      2

c)       3

d)      4

e)       5

f)        ≥ 6

5)      Training program/specialty

6)      Country of practice.

7)      Do you have any formal training in chronic non-cancer pain (CNCP) care in the ED?

a)       If yes, how was this training delivered?

8)      Do you follow any particular clinical practice guidelines regarding CNCP patient care in your ED?

a)       If yes, which guideline(s) do you follow?

9)     How familiar are you in diagnosing various pain syndromes/mechanisms?

a)      Chronic myofascial pain:  1 – Not familiar to 5 – very familiar

b)      Fibromyalgia: 1 – Not familiar to 5 – very familiar

c)       Neuropathic pain:  1 – Not familiar to 5 – very familiar

d)      Inflammatory pain:  1 – Not familiar to 5 – very familiar

10)   I feel that I have adequate training to treat patients with chronic pain

a)      Likert – Not at all (1) to Completely (5)

Caregiver Attitudes towards Pain Patients

11)   A patient’s gender affects how they deal with pain

a)      Likert – Strongly disagree (1) to Strongly agree (5)

12)   Physicians should prescribe analgesics, including opioids for chronic non-cancer pain.

a)      Likert – Strongly disagree (1) to Strongly agree (5)

13)   It is appropriate for chronic pain patients to as for additional analgesics.

a)      Likert – Strongly disagree (1) to Strongly agree (5)

14)   Strong analgesics should only be used for breakthrough pain.

a)      Likert – Strongly disagree (1) to Strongly agree (5)

15)   Patients suffering from chronic pain are usually sufficiently treated for their symptoms.

a)      Likert – Strongly disagree (1) to Strongly agree (5)

16)   I feel comfortable treating patients with chronic pain

a)      Likert – Not at all comfortable (1) to Completely comfortable (5)

17)   I am comfortable prescribing NSAIDs in the ED

a)      Likert – Not at all comfortable (1) to Completely comfortable (5)

18)   I am comfortable prescribing opioids in the ED

a)      Likert – Not at all comfortable (1) to Completely comfortable (5)

19)   I am comfortable recommending non-pharmacologic pain treatments in the ED

a)      Likert – Not at all comfortable (1) to Completely comfortable (5)

20)   I prescribe NSAIDs in the ED

a)      Likert – Never (1) to Often (5)

21)   I prescribe opioids in the ED

a)      Likert – Never (1) to Often (5)

22)   I recommend non-pharmacologic pain treatments in the ED

a)      Likert – Never (1) to Often (5)

23)   What is an acceptable level of pain relief for chronic pain patients presenting with acute pain exacerbation to the ED?

a)      Likert – No pain relief (1) to Complete pain relief (5)

24)   Words and phrases that come to mind when describing a patient with chronic pain are:

25)   A 36-year-old male presents to the ED with a one-month history of frontal headaches. He states that the headaches are 10/10 and causes dizziness with positional changes. He has vomited twice in the last 24 hours due to severe pain. His past medical history is significant for chronic neck and back pain managed on hydromorphone. He states that he has run out of his prescription and was unable to be seen by his physician prior to his ED presentation. His physical exam reveals that he is afebrile. He has no focal neurological deficits. No photophobia or phonophobia. No history of recent trauma. No meningeal signs. No changes in vision. No signs of temporal arteritis. Fundoscopic exam is normal. My perceptions of this patient are: My next step in management would be: I would use the following medications for pain control/I would not offer pain control because:

26)   A 67-year-old female presents to the ED with acute pain from previously diagnosed rheumatoid arthritis. She states that the pain has become unbearable and interferes with her activities of daily living. She uses celecoxib and physiotherapy to help with pain control and appears to be compliant with her treatment plan. The progression of her disease is being followed by a rheumatologist. Physical exam reveals stiff and exquisitely tender metacarpophalangeal (MCP) and proximal interphalangeal (PIP) joints in her hands bilaterally. Swelling is observed in these joints. Boutonniere deformities are also seen. Subluxation of MCPs with an ulnar deviation is seen on x-ray. My perceptions of this patient are: My next step in management would be: I would use the following medications for pain control/I would not offer analgesics because:

27)   A 23-year-old female with fibromyalgia presents to the ED with intractable pain. Her pain is managed with NSAIDs from her primary care physician and over-the-counter acetaminophen. Chart review reveals that she has presented to the ED six times over the last four weeks due to pain. Each time, she was discharged on a short course of opioids. She states that she has not followed up with her PCP due to a poor physician-patient relationship stemming from her chronic pain and is in the process of finding a new physician. However, the prior ED prescriptions have been effective at managing her pain. She is requesting another prescription of her previous ED medication. Her physical exam is unremarkable, except severe widespread pain. My perceptions of this patient are: My next step in management would be: I would use the following medications for pain control/I would not offer analgesics because:

28)   A 45-year-old male presents to the ED with acute exacerbation of his lower back and leg pain. He describes the pain as 10/10 and radiates down the posterior aspect of the leg. This occurred as he was lifting a heavy object. His past medical history is significant for degenerative disc disease at the L5 level and sciatica. He has been managing with a transdermal fentanyl patch and short-acting opioids for breakthrough pain. On review of systems, he states that he has noticed progressively worsening shortness of breath over the last three months. He has a 15 pack/year smoking history. Physical exam reveals extreme tenderness to palpation over the lumbar spine at the L5 level. Straight leg raise and foot dorsiflexion also reproduce the pain. My perceptions of this patient are: My next step in management would be: I would use the following medications for pain control/I would not offer analgesics because:
